# A simple one-step PCR assay for SNP detection

**DOI:** 10.17912/micropub.biology.000399

**Published:** 2021-06-11

**Authors:** Jian Chen, Tim Schedl

**Affiliations:** 1 Department of Genetics, School of Medicine, Washington University in St. Louis, Missouri 63110.

## Abstract

Polymerase Chain Reaction (PCR) is a powerful tool to detect natural variation or experimentally introduced variation in research and clinical settings and a widely-used method for genotyping. Single nucleotide polymorphisms (SNP) detection is challenging by PCR as the variant and wild type alleles differ by only one nucleotide. Traditional methods to detect SNPs, including Sanger sequencing and commercial kits, are usually time-consuming. Here we describe a simple primer design strategy that enables specific variant detection through regular one-step PCR. The strategy employs the differential efficiency of genomic PCR using a primer that has a single mismatch with the chromosome that contains the SNP to be detected (typically the variant allele) versus two mismatches with the corresponding alternative allele (typically the wild type allele). To date, we have successfully employed this approach to detect more than 20 SNPs. The simplicity and robustness of the approach allows rapid application to legacy mutations as well as newly discovered or generated SNPs.

**Figure 1. Novel primer design strategy to detect single nucleotide polymorphisms (SNPs) by PCR.**
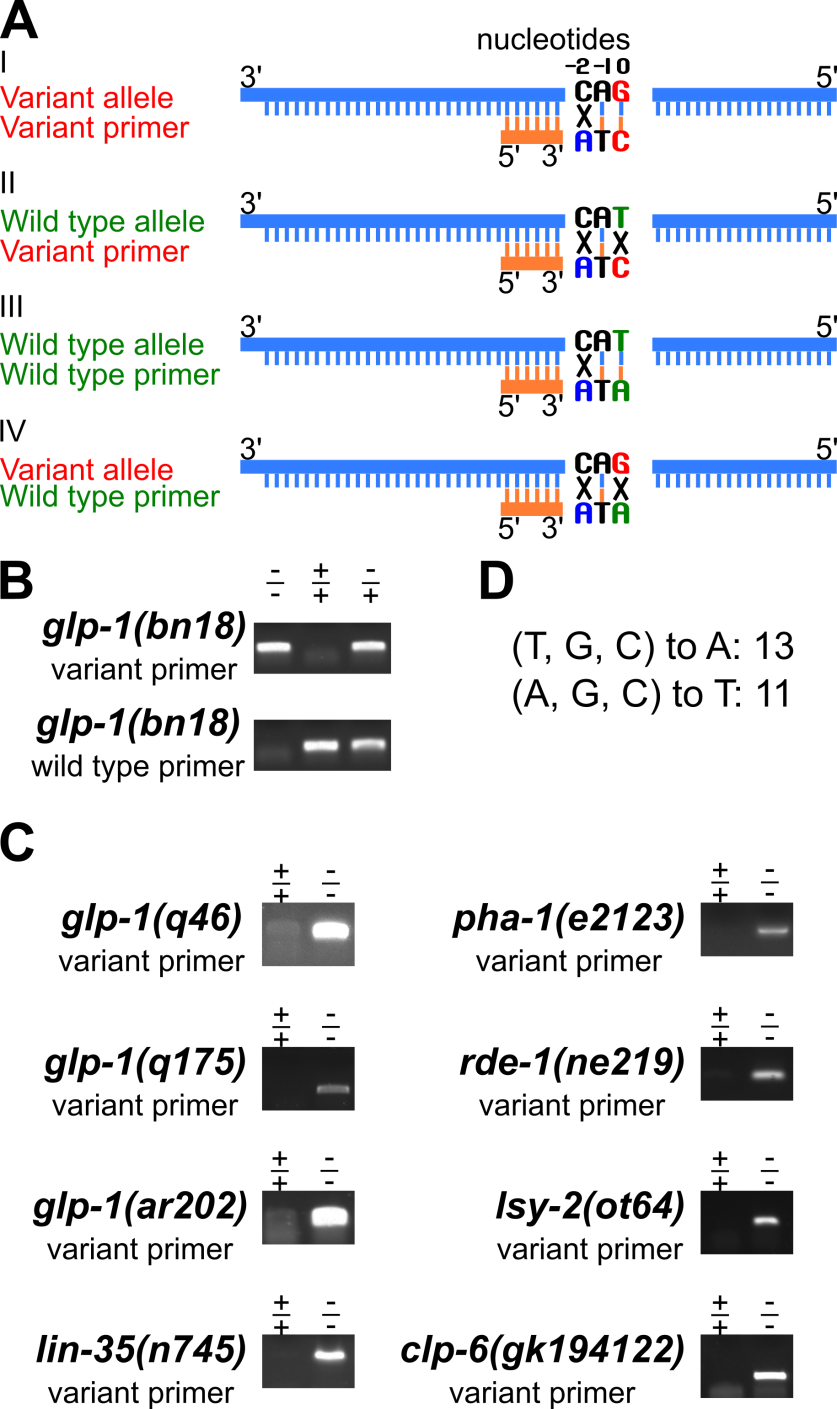
(A) Schematic showing the primer design strategy to discriminate between the variant and wild type alleles through PCR (compare I and II, III and IV). The SNP residue is denoted as position 0. G, in red, is the variant allele, while T, in green, is the wild type allele. The forward PCR primer was designed to specifically detect either the variant or wild type allele, with the 3’ end starting at the SNP residue (G or T at position 0). For the allele to be detected, there is no mismatch at position 0, while for the other allele, there is a mismatch (I versus II, and III versus IV). To increase the specificity for the allele to be detected by PCR, a second change (C to A in these examples, in blue) was introduced at the -2 position, two nucleotides upstream of the SNP position. For examples I and II, the variant forward primer can bind more efficiently to the variant allele (one mismatch in I) than the wild type allele (two mismatches in II), resulting in a more robust PCR amplification of the variant allele. Similarly for examples III and IV, the wild type forward primer can detect the wild type allele (shown in III) more effectively than the variant allele (shown in IV). “X” indicates mismatch, **|** indicates pairing. The reverse PCR primer was identical for both the variant and wild type allele (see Table 1) and has generated from 200 to 400 nucleotide amplification products. (B & C) Representative agarose gel images of single worm PCR products amplified with primers designed to specifically detect alleles of the indicated genes, either the variant or the wild type allele (Table 1). The genotype is shown above the corresponding lane, with – indicating the variant allele and + indicating the wild type allele. (B) Gel images from primers to detect the *glp-1(bn18)* mutant allele or the wild type allele. Single worm lysis of animals with the indicated genotype were sampled twice, and PCR was performed with variant primer (upper image) or the wild type primer (lower image). -/-, *glp-1(bn18)* homozygote; +/+, N2 wild type; -/+, *glp-1(bn18)/ hT2::gfp[bli-4(e937) let-?(q782) qIs48]*. (C) Gel images showing specific detection of the variant allele for six genes and eight variants. -/-, indicates the relevant gene-variant homozygote; +/+, N2 wild type. (D). The distribution of nucleotide changes introduced at the -2 position from a total of 24 primers. The nucleotide at -2 position was either changed to an A or a T.

## Description

Single nucleotide polymorphisms (SNPs) are the most common genetic variation between natural populations of a species, are the frequent cause of phenotypes observed in organisms from forward genetic chemical mutagenesis screens, and are employed to probe the functional consequences of missense and noncoding changes made by CRISPR/Cas9 gene editing (Brenner, 1974; Okamoto *et al.*, 2019; Thompson *et al.*, 2013; Zhao *et al.*, 2014). For model organisms, SNP genotyping is essential for strain construction and is an important part of strain validation necessary for research rigor and reproducibility. This is particularly the case if the SNP does not have a known phenotype, does not have an easy way to score phenotype, or the phenotype is masked by epistasis. Additionally, laboratories are typically working with multiple genes, often examining multiple alleles, which could be legacy mutations or newly discovered/generated variants. Therefore, a simple SNP detection approach is desirable to allow rapid genotyping. Furthermore, the approach should be nimble in the sense of being applicable to any gene, potentially any SNP, and the generation of a new assay and its execution being straightforward and occurring in a short time frame. Here we describe a simple system for single worm genotyping. The strategy employs the differential efficiency of genomic PCR using a primer that has a single mismatch with the chromosome that contains the SNP to be detected (typically the variant allele) versus two mismatches with the corresponding alternative allele (typically the wild type allele). The genotype of a worm is deduced from two samples of the single worm lysis, where one PCR reaction uses a primer with mismatches to specifically detect the variant and not the wild type allele and the other PCR reaction uses a primer with mismatches to specifically detect the wild type allele and not the variant.

Key to the approach is a robust PCR method to detect one allele but not the other at a SNP residue (Figure 1A). A forward and a reverse primer are needed for each PCR reaction. We have designed the forward primer to more efficiently amplify one of the alleles. There are two important features of the forward primer. First, the 3’ nucleotide of the primer is at the SNP, denoted as 0 position, and pairs with the SNP residue to be detected but is mismatched with the other allele residue. Therefore the variant and wild type primers have different bases at their 3′ end. Second, two bases upstream of the SNP position, denoted as -2 position, an additional change was introduced that is a mismatch with both the variant and wild type alleles (Figure 1A, in blue in examples I to IV), to further discriminate between the two alleles in PCR. The reverse primer is identical in both cases. As shown in Figure 1A examples I to IV, the variant allele “G” and complement “C” are highlighted in red, and the wild type allele “T” and complement “A” are in green (Figure 1A). The variant primer has one mismatch relative to the variant allele (Fig. 1A, example I), compared to two mismatches relative to the wild type allele (Fig. 1A, example II). Therefore the variant primer can selectively amplify the variant allele. Similarly, the wild type primer can specifically detect the wild type allele (Figure 1A, examples III and IV). We have successfully applied this primer design strategy to detect the *glp-1(bn18)* variant allele (Kodoyianni *et al.*, 1992) and the wild type allele through PCR (Figure 1B; genomic position III: 9098493, WormBase). By sampling the single worm lysis twice and performing separate PCR reactions with primers for the variant and the wild type allele, the genotype at the *glp-1* *bn18* residue is determined. As shown in Figure 1B, the variant primer preferentially amplifies the variant allele, with minimal background signal detected within wild type sample. Similarly, the wild primer preferentially amplifies the wild type allele, with minimal background signal detected within the homozygous *bn18* variant sample. Gel images for an additional eight SNPs with variant specific primers are presented in Figure 1C. A summary of the nucleotide changes introduced at -2 position is shown in Figure 1D. To date, we have applied this approach to detect more than 20 SNPs, including additional *glp-1* alleles (Kodoyianni *et al.*, 1992; Pepper *et al.*, 2003) and other genes (Johnston & Hobert, 2005; Lu & Horvitz, 1998; Mani & Fay, 2009; Tabara *et al.*, 1999; Thompson *et al.*, 2013). In summary, the primer design strategy presented here can be easily applied to legacy and newly identified SNPs, reduces time/effort and reagent costs, significantly simplifying SNP detection and genotyping by PCR in *C. elegans*, and likely in other systems.

## Methods

All primers designed to have Tm between 58 °C to 61 °C. GoTaq DNA Polymerase (Promega, cat#M3008) was used in all PCR reactions described, following the manufacturer’s instructions. The single worm lysis PCR procedure is as previously described (Barstead *et al.*, 1991; Williams *et al.*, 1992). All PCR used the same cycling program. Initial denaturation of genomic DNA occurred at 94 °C for 2 minutes, 35 repeats of following: denature at 94 °C for 20 seconds, annealing at 55 °C for 20 seconds, and extension at 72 °C for 30 seconds. Final extension occurred at 72 °C for 2 minutes. No special treatment was required before the PCR samples were loaded on an agarose gel.

## Reagents

Table 1: primers used in this study

**Table:** 

**Primer_name**	**Primer_seq**
650_glp-1_bn18_variant_F	gatgaattggaccggaatggtatga*A*t**A**
649_glp-1_bn18_wildtype_F	gatgaattggaccggaatggtatga*A*t**G**
190_glp-1_bn18_R	agagctgttcgtcctttatacttgt
20_glp-1_q46_variant_F	gggcaaagaccattctccaaat*A*t**T**
21_glp-1_q46_R	ctccatcgcctcgtctttcaatac
766_glp-1_q175_variant_F	ggaaaatccggtcgatattgtg*T*a**T**
767_glp-1_q175_R	gcagtgtggtctctgtagtggaa
630_glp-1_ar202_variant_F	cagggtattgacatttggagaatggtcttt*A*c**T**
260_glp-1_ar202_R	gagccacttggagtataatgacgatg
674_lin-35_n745_variant_F	ccaaatgacattgttactggtgca*A*g**A**
675_lin-35_n745_R	tgtcaagcatttcagcaacgga
684_pha-1_e2123_variant_F	taacttgatgaacatcggtaatcatac*T*g**T**
685_pha-1_e2123_R	cttaatgcccttgcaccgtagt
646_rde-1_ne219_variant_F	gtggcttctcatgaacttcaagatg*A*t**T**
192_rde-1_ne219_R	aaatcggacagaggaagaaatgca
692_lsy-2_ot64_variant_F	gatctgtgtgtatcactgcatg*A*t**A**
693_lsy-2_ot64_R	ctgaagaagatgagatggtggaagg
1149_clp-6_gk194122_variant_F	ggcagtcgatcatcaattactacatca*T*c**T**
1150_clp-6_gk194122_R	ccttgttgggtcatttccacgt

Notes to the table 1: The bold nucleotide at 3’ end of the forward primer denotes either the variant allele or the wild type allele. The italicized nucleotide denotes mismatch change that was introduced two bases upstream of the SNP site. F forward primer; R reverse primer.
